# Amyloid-Beta (Aβ) D7H Mutation Increases Oligomeric Aβ42 and Alters Properties of Aβ-Zinc/Copper Assemblies

**DOI:** 10.1371/journal.pone.0035807

**Published:** 2012-04-30

**Authors:** Wei-Ting Chen, Chen-Jee Hong, Ya-Tzu Lin, Wen-Han Chang, He-Ting Huang, Jhih-Ying Liao, Yu-Jen Chang, Yi-Fang Hsieh, Chih-Ya Cheng, Hsiu-Chih Liu, Yun-Ru Chen, Irene H. Cheng

**Affiliations:** 1 Institute of Brain Science, National Yang Ming University, Taipei, Taiwan; 2 Division of Psychiatry, National Yang Ming University, Taipei, Taiwan; 3 Institute of Clinical Medicine, National Yang Ming University, Taipei, Taiwan; 4 Division of Neurology, National Yang Ming University, Taipei, Taiwan; 5 Brain Research Center, National Yang Ming University, Taipei, Taiwan; 6 Genomics Research Center, Academia Sinica, Taipei, Taiwan; 7 Taiwan International Graduate Program in Molecular Medicine, National Yang-Ming University and Academia Sinica, Taipei, Taiwan; 8 Department of Psychiatry, Taipei Veterans General Hospital, Taipei, Taiwan; 9 Department of Neurology, Taipei Veterans General Hospital, Taipei, Taiwan; Mental Health Research Institute of Victoria, Australia

## Abstract

Amyloid precursor protein (APP) mutations associated with familial Alzheimer's disease (AD) usually lead to increases in amyloid β-protein (Aβ) levels or aggregation. Here, we identified a novel APP mutation, located within the Aβ sequence (Aβ_D7H_), in a Taiwanese family with early onset AD and explored the pathogenicity of this mutation. Cellular and biochemical analysis reveal that this mutation increased Aβ production, Aβ42/40 ratio and prolonged Aβ42 oligomer state with higher neurotoxicity. Because the D7H mutant Aβ has an additional metal ion-coordinating residue, histidine, we speculate that this mutation may promote susceptibility of Aβ to ion. When co-incubated with Zn^2+^ or Cu^2+^, Aβ_D7H_ aggregated into low molecular weight oligomers. Together, the D7H mutation could contribute to AD pathology through a “double punch” effect on elevating both Aβ production and oligomerization. Although the pathogenic nature of this mutation needs further confirmation, our findings suggest that the Aβ N-terminal region potentially modulates APP processing and Aβ aggregation, and further provides a genetic indication of the importance of Zn^2+^ and Cu^2+^ in the etiology of AD.

## Introduction

**Figure 1:**
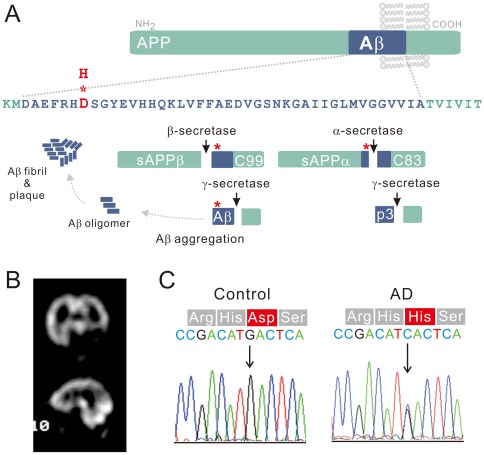
A novel mutation leads to an aspartate to histidine substitution at the N-terminus of Aβ. (A) The upper part of the diagram presents the Aβ42 sequence with the location of the D7H mutation (red). As shown in the lower part of the diagram, processing of APP occurs via two pathways. Nonamyloidogenic processing of APP by á-secretase produces the C83 and sAPPα fragments; amyloidogenic processing of APP by â-secretase produces the C99 and sAPPβ fragments. Aβ is generated through subsequent cleavage of C99 by γ-secretase. (B) SPECT images of the index patient indicate hypoperfusion in the bilateral parietal cortices and the left temporal cortex. (C) Direct sequencing of APP exon 16 PCR products derived from the patient and from healthy controls revealed a GAC-to-CAC nucleotide substitution in Aβ region of the patient's APP gene (in 678^th^ amino acid using APP770 numbering or in 7^th^ amino acid using Aβ numbering).

Alzheimer's disease (AD) is characterized neuropathologically by progressive brain deposition of the amyloid β peptide (Aβ), which is generated by proteolytic cleavage of amyloid precursor protein (APP) by β- and γ-secretases (Fig. 1A). The two most common Aβ variants have 40 (Aβ40) or 42 (Aβ42) amino acids. The abnormal aggregation and accumulation of neurotoxic Aβ have been proposed as the primary driving force for AD in the amyloid hypothesis [1].

Aβ aggregation undergoes multiple pathways with a variety of intermediates/oligomers formation. The current notion is that low molecular weight (LMW) assemblies such as soluble oligomers and protofibrils, but not fibril, are the primary toxic structures of Aβ [2], [3]. However, due to the highly dynamic nature of Aβ assemblies and the technical limitation, biochemical features of toxic Aβ aggregates remain unclear [4].

Mutations in the APP gene lead to the early onset familial AD. Most APP mutations are concentrated either around or within the Aβ domain. APP mutations at the secretase cleavage sites accelerate the production of Aβ, particularly the highly pathogenic Aβ42 [5], [6], [7], [8], [9]. Mutations clustered within the 21^st^–23^rd^ residues of Aβ involve enhancing Aβ aggregation, delaying Aβ elimination or increasing Aβ production [9], [10], [11], [12], [13]. Mutations located at Aβ N-terminus, including the English (H6R) and Tottori (D7N) mutations, have been shown to enhance fibril formation without altering Aβ production [14]. Several potential therapeutic strategies aimed at reducing Aβ production, inhibiting Aβ aggregation, and speeding Aβ removal are being developed [15].

Metal ions, especially Zn^2+^ and Cu^2+^, have been shown to abnormally accumulate in the amyloid plaques of patients with AD [16]. The interplay of metal-Aβ interaction has been strengthened recently [17]. Metal ions with redox activity, such as Cu^2+^ and Fe^3+^, induce free radicals through the formation of Aβ-ion complex [18]. Zn^2+^ and Cu^2+^ are known to bind the histidine residues at Aβ N-terminus [19], [20]. The metal chelation therapy is now a potential treatment for AD and undergoing clinical phase IIb trial [21], [22]. However, disruption of ion homeostasis in the central nervous system by the use of metal chelator may further deplete the essential metal ions and cause negative impact on the disease progress [23]. Therefore, to specify the features of the Aβ-ion complex could help to improve the pharmacological design.

Here, we report a novel intra-Aβ mutation (D7H) in a Taiwanese family with early onset AD. Because the number of patients is limited, we explored the pathogenicity of this mutation with experimental approaches. we propose this mutation is probable pathogenic because the D7H mutation resulted in increased levels total Aβ, in a higher Aβ42/40 ratio and in the formation of Aβ40 fibrils while prolonged Aβ42 oligomers state with higher toxicity. Furthermore, we speculated that the appearance of one more histidine at the 7^th^ residue of mutant Aβ may enhance susceptibility to the effect of Zn^2+^ or Cu^2+^. Our study reveals that this mutation increased the binding of Zn^2+^ and Cu^2+^ and promoted the formation of ion-induced Aβ oligomers with altered morphology. Together, our clinical and experimental results suggest a pathogenic role of the D7H mutation in familial AD. We also provide a “genetic hint” for the studies in the metal as etiology in AD.

## Results

### Clinical description and genetic analysis

We identified a 53 year-old female AD patient who had multiple family members affected with memory impairment before age 65 (Fig. S1A). The index patient had been showing progressive memory impairment, slurred speech, persecutory delusions, self-talking and inability to dress herself since age 51. She was restless and asked the same questions repeatedly during the clinical examinations. The scores of the mini mental status examination and the Wechsler Adult Intelligence Scale were 13 and 62, respectively. The computed tomography (CT) scans revealed diffuse prominent cerebral fissures, cisterns and sulci. The Tc-99 m HMPAO single photon emission computed tomography (SPECT) scans showed hypoperfusion in the bilateral parietal and left temporal cortices (Fig. 1B). Diffuse background slow waves (6–7 Hz) were noted by electroencephalography. The results of blood biochemistry tests for liver function, renal function, thyroid function, anemia and syphilis were all within normal limits (Fig. S1B). Diagnosis of probable AD was made according to the NINCDS–ADRDA criteria. Mutation analysis was done by directly sequencing PCR-amplified coding exons of *PSEN1*, *PSEN2* and *APP*. Sequencing revealed a G→C nucleotide substitution in the *APP* gene, resulting in an aspartate to histidine mutation at 7^th^ position of Aβ (D678H using APP770 numbering or D7H using Aβ numbering, Fig. 1C). This mutation has never been reported and was not found in 100 unrelated healthy controls and 100 Chinese AD patients.

Due to the limited number of patients, we tried to determine the pathogenicity of this mutation by functional analysis. Both cells expressing human APP and synthetic Aβ peptides were used to explore the levels of Aβ production, Aβ42/40 ratio and Aβ aggregation process.

### The D7H mutation on APP enhances amyloidogenic cleavage and increases the Aβ42/40 ratio

**Figure 2:**
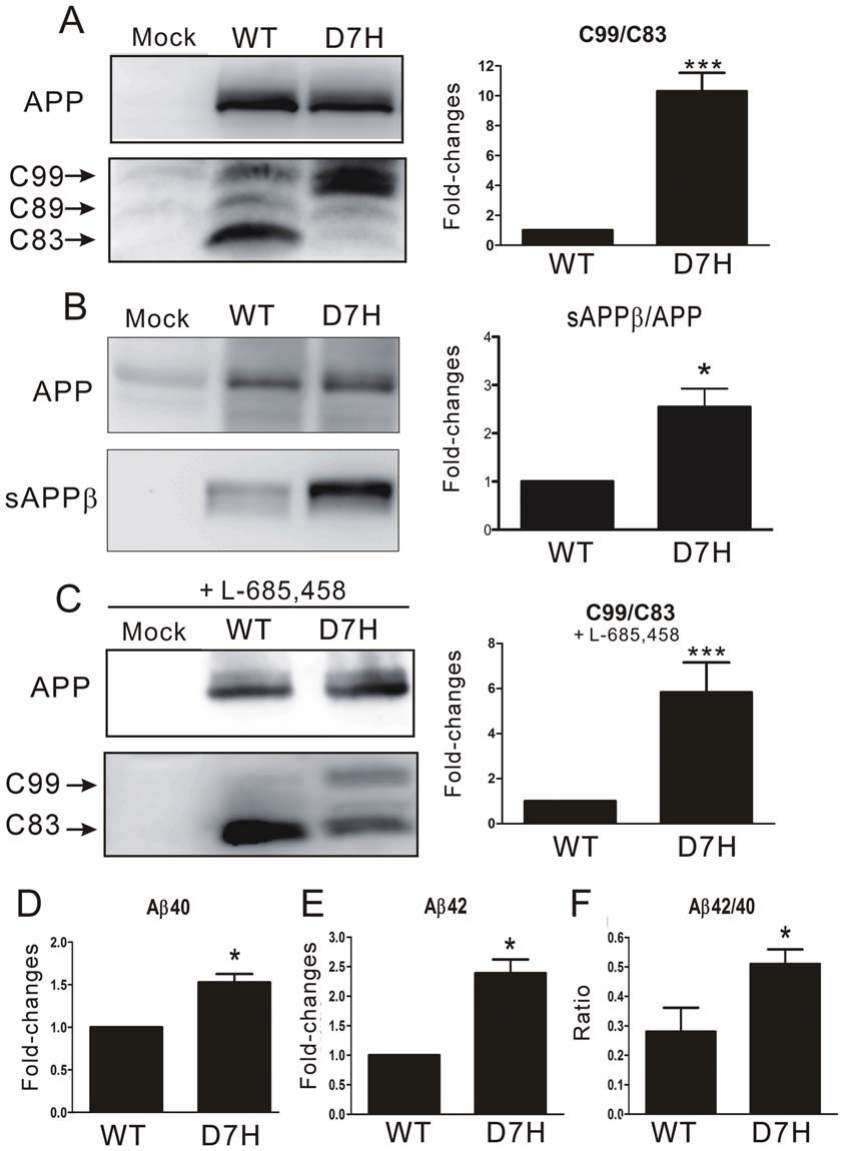
The D7H mutation increases Aβ production and the Aβ42/40 ratio. (A–C) Western blots were used to monitor the levels of full length APP, the C99 and C83 fragments (A, C) and the sAPPβ fragment (B) in HEK293 cells transfected with empty vector (mock), wt APP or D7H mutant APP cDNAs. Densitometric analysis on the right showed a significant increase of the C99/C83 ratio and sAPPβ in cells expressing D7H mutant APP in both the absence (A, B) and presence (C) of γ-secretase inhibitor L-685,458. (D–F) ELISA showed significantly higher fold-change of Aβ40/APP (D), Aβ42/APP (E) and Aβ42/40 (F) in the conditioned media of D7H mutant APP transfected cells. All the data were normalized to data from wt APP-expressing cells (set as 1) in 3 independent experiments (n = 3 per experiment) and presented as mean ± SEM. ***P<0.001, *P<0.05 by one-way ANOVA and Turkey post-test.

In the non-amyloidogenic pathway, cleavage of APP within the Aâ region by α-secretase generates a secreted N-terminal fragment α (sAPPα) and an 83 amino acid C-terminal fragment (C83) and, thus, precludes Aâ formation. In the amyloidogenic pathway, cleavage of APP by β-secretase generates a secreted N-terminal fragment β (sAPPβ) and a 99 amino acid C-terminal fragment (C99) (Fig. 1A) [24]. To elucidate whether the D7H mutation shifts the balance between these two pathways, we transiently transfected human embryonic kidney (HEK293) cells with either human wild type (wt) or D7H mutant APP cDNA. The transfection efficiency of both wt and D7H mutant APP are both ∼20% (Fig. S2A) and protein expression levels for both wt and D7H mutant APP were similar (Fig. S2B). In addition, there is no significant difference in mature/immature APP ratio (Fig. S2C). The levels of full length APP, the α-secretase cleavage product C83, and the β-secretase cleavage products C99 and sAPPβ were measured by the Western blot. In cells expressing wt APP, the C83 fragment (∼10 kDa) was the predominant species detected. In cells expressing D7H mutant APP, the C99 fragment (∼12 kDa) was the predominant species detected (Fig. 2A). Densitometric analysis revealed that the ratio of C99/C83 in cells expressing D7H mutant APP was 10.3 fold higher than in cells expressing wt APP (Fig. 2A). Both cells had no significant difference on the level of β′-cleavage product C89 (∼11 kDa). Besides, the level of sAPPβ was significant higher in the conditioned media of cells expressing D7H mutant APP than that of cells expressing wt APP (Fig. 2B). Thus, the D7H mutation may shift APP processing from the non-amyloidogenic to the amyloidogenic cleavage pathway.

The higher C99/C83 ratio may be due to either increased C99 production by β-secretase or to delayed C99 removal by γ-secretase. To distinguish between these two possibilities, we inhibited γ-secretase activity by adding 1 μM L-685,458 to the media for 24 h. The ratio of C99/C83 in cells expressing D7H mutant APP was 5.8 fold higher than in cells expressing wt APP when treated with the inhibitor (Fig. 2C) but was lower than in cells not treated with inhibitor (10.3 fold). Therefore, both the production and cleavage of C99 were altered by the D7H mutation.

We next examined whether the D7H mutation alters the extracellular and intracellular Aβ levels or the Aβ42/40 ratio. HEK293 cells and conditioned media were both collected at 48 h after APP transfection. Aβ levels were measured by enzyme-linked immunosorbent assay (ELISA) and normalized to total APP level. The conditioned media of D7H mutant APP transfected culture had 1.5 fold higher extracellular Aβ40 level and 2.4 fold higher Aβ42 level compared to that of the wt APP transfected culture (Fig. 2D, E). Among all variants of Aβ, Aβ42 is especially prone to misfolding and aggregating into toxic assemblies [7], [8]. We found that D7H mutant APP transfected culture had a significantly higher ratio of extracellular Aβ42/40 than the wt APP transfected culture (Fig. 2F). The accumulation of intracellular Aβ may also contribute to the pathogenesis of AD. However, we did not find significant differences in intracellular Aβ levels or in the intracellular Aβ42/40 ratio between wt APP and D7H mutant APP expressing cells (Fig. S3).

### The D7H mutation switches the Aβ aggregation process

To investigate the effect of the D7H mutation on Aβ aggregation, we monitored the kinetics of fibril formation, the size distribution and the morphology of Aβ_wt_ and Aβ_D7H_ assemblies by the thioflavin T (ThT) assay, Western blot, and transmission electron microscopy (TEM). Synthetic Aβ peptides were dissolved in HFIP-DMSO for the Western blot, toxicity and TEM experiments and in guanidine hydrochloride (GdnHCl) for the ThT assay because GdnHCl-denatured Aβ allows us to better distinguish the kinetics of the early stages of aggregation.

#### The D7H mutation promotes Aβ40 fibril formation

**Figure 3:**
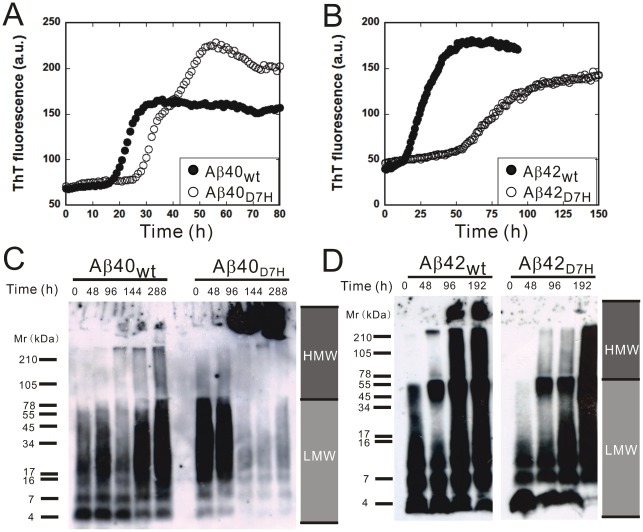
The D7H mutation promotes Aβ40 HMW assemblies and Aβ42 LMW assemblies formation. Lyophilized Aβ was prepared in GdnHCl for the ThT assay (A, B) or in HFIP-DMSO for Western blot (C, D), and samples were collected at indicated times. (A, B) The ThT assay was applied to monitor the kinetics of β-sheet formation for Aβ40_wt_ (A,•), Aβ40_D7H_ (A,○), Aβ42_wt_ (B,•) and Aβ42_D7H_ (B,○). Data were averaged from 3–4 independent experiments (n = 3 per experiment). (C) Aβ40 and (D) Aβ42 samples were fixed by PICUP and examined by Western blot to analyze the size distribution of assemblies during aggregation.

**Figure 4:**
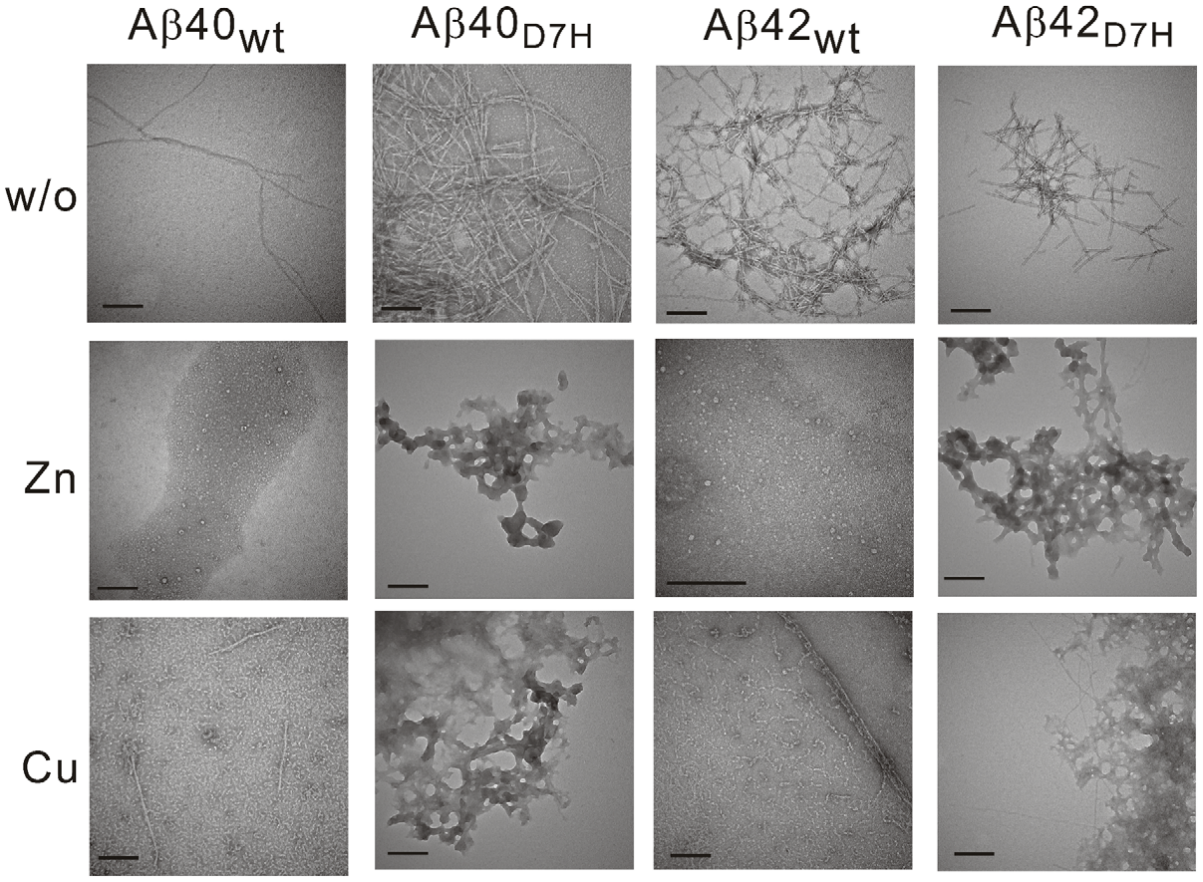
Aβ morphology in the presence or absence of metal ions was revealed by TEM. Lyophilized Aβ was prepared in HFIP-DMSO. After 264–312 h of incubation in either the presence or absence of Zn^2+^ or Cu^2+^, the Aβ samples were stained by 2% uranyl acetate and monitored by TEM. In the presence of ions, the Aβ_D7H_ peptides were predominantly amorphous morphology but not protofibrils as Aβ_wt_. Scale bar: 200 nm.

In the ThT analysis of fibrillization kinetics, the lag time of the initiation of fibril formation for Aβ40_D7H_ (∼28 h) was longer than that for Aβ40_wt_ (∼18 h). However, in the saturation phase, the ThT fluorescence intensity of Aβ40_D7H_ was ∼1.5 fold higher than that of Aβ40_wt_ (Fig. 3A). In order to analyze the size distribution of Aβ assemblies by Western blot, we used the photo-induced cross-linking of unmodified protein (PICUP) approach to “freeze” the Aβ assemblies at indicated time points [25]. At the initial time point, both Aβ40_wt_ and Aβ40_D7H_ were predominantly present as low molecular weight (LMW) assemblies (Fig. 3C). After 96 h of incubation, more Aβ40_D7H_ than Aβ40_wt_ aggregated into high molecular weight (HMW) assemblies. Here, we defined the Aβ assemblies that can be separated by 15% Tricine-PAGEs as LMW (usually below 78 kDa) while the larger Aβ assemblies retaining in stacking gel as HMW. Using TEM, we observed more fibrillar structures in the Aβ40_D7H_ assemblies and more oligomeric or protofibrillar structures in the Aβ40_wt_ assemblies after 312 h of incubation (Fig. 4). All of these experiments indicate that the D7H mutation slightly delays Aβ nucleation and promotes the formation of Aβ40 HMW assemblies and fibrils.

#### The D7H mutation promotes Aβ42 LMW assembly formation

Next, we studied the effect of the D7H mutation on Aβ42 aggregation using the same approaches as described above for Aβ40. Unexpectedly, the D7H mutation did not promote the formation of Aβ42 fibrils but rather prolonged the duration of Aβ42 oligomers.

In the ThT analysis of fibrillization kinetics, the lag time of the initiation of fibril formation for Aβ42_D7H_ (∼58 h) was longer than that for Aβ42_wt_ (∼18 h). In the saturation phase, the ThT fluorescence intensity of Aβ42_D7H_ was ∼1.35 fold lower than that of Aβ42_wt_ (Fig. 3B). In the Western blot analysis, Aβ42_wt_ quickly aggregated into HMW assemblies after 48 h while Aβ42_D7H_ remained in LMW assemblies until 192 h when it gradually aggregated into HMW assemblies (Fig. 3D). Using TEM, we observed more oligomeric or protofibrillar structures in the Aβ42_D7H_ assemblies and more fibrillar structures in the Aβ42_wt_ assemblies after 312 h of incubation (Fig. 4). All of these results indicate that the D7H mutation results in Aβ42 remaining in LMW assemblies longer and in reduced HMW fibril formation.

Considering that the GdnHCl in our Aβ preparation might affect the assembly state [4], the results of the ThT assay were also confirmed by preparing Aβ in HFIP-DMSO (Fig. S4A, B). In this condition, the D7H mutation also increased Aβ40 fibril formation and decreased Aβ42 fibril formation. Considering the possibility that artificial assemblies may be induced by PICUP, Western blot analysis without PICUP was also performed (Fig. S4C, D). These results also confirmed that the D7H mutation promotes the formation of Aβ40 HMW assemblies and prolongs the time Aβ42 remains in LMW assemblies.

### The D7H mutation promotes Aβ42 neurotoxicity

**Figure 5:**
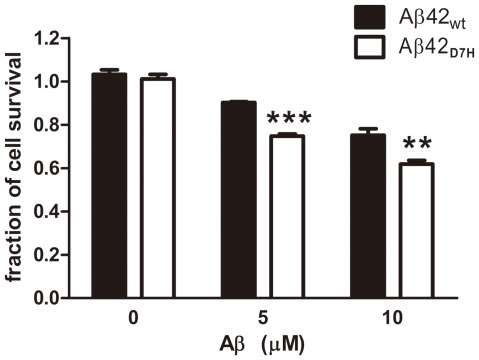
The D7H mutation enhances the neurotoxicity of Aβ42. The neurotoxicities of Aβ42_wt_ and Aβ42_D7H_ were estimated by the MTT assay. SH-SY5Y cells were treated with Aβ42_wt_ or Aβ42_D7H_ at a final concentration of 0, 5, or 10 μM for 48 h. Cell survival was determined by normalizing OD570 readings to those of cells not treated with Aβ42 (set as 1) in 3 independent experiments (n = 8 per experiment) and is presented as mean ± SEM. ***P<0.001, **P<0.01 vs. Aβ42_wt_ by ANOVA.

Because the oligomers are generally considered to be the more neurotoxic Aβ assembly state, the increase of Aβ42_D7H_ oligomers may promote neurotoxicity. To determine the effect of the D7H mutation on the neurotoxicity of Aβ42 oligomers, synthetic Aβ42_wt_ and Aβ42_D7H_ were prepared using HFIP-DMSO and incubated at 4°C for 24 h. The neurotoxicity of these Aβ42 assemblies on SH-SY5Y human neuroblastoma cells was measured using the MTT assay. After 48 h of co-incubation with either 5 μM or 10 μM Aβ42_D7H_, SH-SY5Y cells had significantly lower survival rates than cells incubated with Aβ42_wt_ (Fig. 5). Our results indicate that the D7H mutation promotes neurotoxicity of Aβ42.

Together, this mutation increased Aβ production, Aβ42/Aβ40 ratio, and prolonged Aβ?2 oligomer state with higher neurotoxicity. Therefore, we propose to classify the D7H mutation as probable pathogenic according to the algorithm proposed previously [26].

### The D7H mutation alters the biochemical features of ion-induced Aβ assemblies

Histidines at the 6^th^, 13^th^ and 14^th^ residues of Aβ are important for the peptide's interaction with the metal ions, which can also affect Aβ aggregation [19], [20]. We speculated that the appearance of one more histidine at the 7^th^ residue of Aβ_D7H_ may enhance susceptibility to the effect of Zn^2+^ or Cu^2+^ on Aβ aggregation. To explore this speculation, we incubated Aβ_wt_ or Aβ_D7H_ with Zn^2+^ or Cu^2+^ to observe the kinetics of fibril formation, size distribution, and morphology of the respective Aβ assemblies.

**Figure 6:**
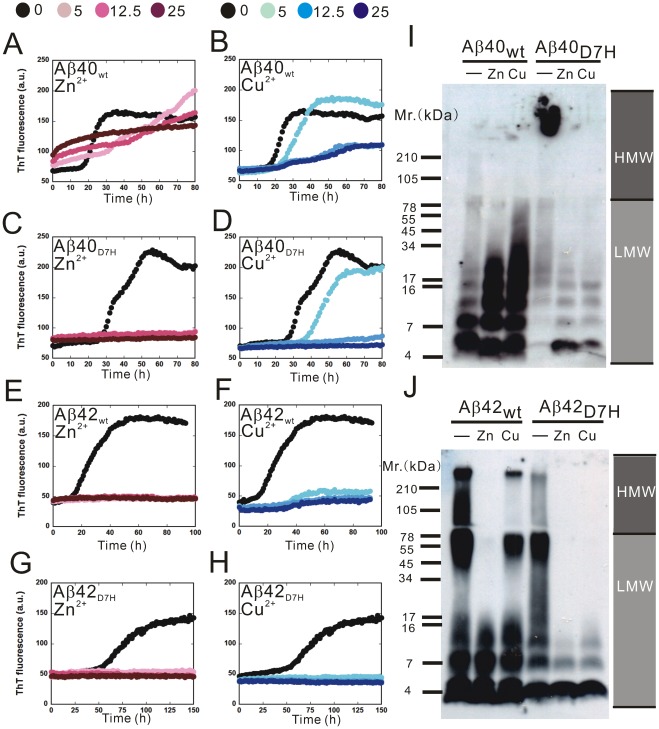
The D7H mutation shifts Zn^2+^ and Cu^2+^-induced assemblies toward smaller oligomers with fewer fibrils. (A–H) 25 μM Aβ was incubated with 25 μM ThT in Tris buffer containing 0 μM (black), 5 μM (light color), 12.5 μM (medium color) or 25 μM (dark color) of Zn^2+^ (red) or Cu^2+^ (blue). (A) Aβ40_wt_ + Zn^2+^, (B) Aβ40_wt_ + Cu^2+^, (C) Aβ40_D7H_ + Zn^2+^, (D) Aβ40_D7H_ + Cu^2+^, (E) Aβ42_wt_ + Zn^2+^, (F) Aβ42_wt_ + Cu^2+^, (G) Aβ42_D7H_ + Zn^2+^, (H) Aβ42_D7H_ + Cu^2+^. (I–J) 25 μM Aβ40 (I) and Aβ42 (J) were co-incubated with 25 μM Zn^2+^ or Cu^2+^ for 114 h, fixed by PICUP and examined by Western blot to analyze the size distribution.

For the ThT assay, Aβ was incubated with Zn^2+^ or Cu^2+^ in 1∶1, 2∶1 and 5∶1 (Aβ: metal ion) ratios for 80–150 h. We found that Zn^2+^ accelerated while Cu^2+^ prolonged initiation of Aβ40_wt_ aggregation as we reported previously [27]. Both ions had stronger inhibitory effects on fibril formation in Aβ40_D7H_ than in Aβ40_wt_ in a dose dependent manner. At a 1∶1 ratio with Zn^2+^, the ThT intensity in the saturation phase of Aβ40_wt_ was 10% lower than that of the no ion control while Aβ40_D7H_ was 90% lower than that of the no ion control (Fig. 6A, C). At a 1∶1 ratio with Cu^2+^, the ThT intensity of Aβ40_wt_ was 50% lower than that of the no ion control while Aβ40_D7H_ was ∼100% lower than that of the no ion control (Fig. 6B, D). These 2 ions also had stronger inhibitory effects on Aβ42 fibril formation for Aβ42_D7H_ than for Aβ42_wt_. Cu^2+^ inhibited fibril formation in Aβ42_D7H_ to a greater extent than in Aβ42_wt_, but Zn^2+^-induced inhibition was similar for Aβ42_wt_ and Aβ42_D7H_ in the ThT assay (Fig. 6E–H).

In the Western blot analysis, Aβ was incubated with Zn^2+^ or Cu^2+^ at a 1∶1 ratio for 144 h. We found that Aβ40_wt_ aggregated into mostly LMW assemblies with or without ions. However, when Aβ40_D7H_ was co-incubated with Zn^2+^ or Cu^2+^_,_ we observed fewer HMW assemblies and more LMW assemblies than in the no ion control (Fig. 6I). Similar to our findings in figure 3D, both Aβ42_wt_ and Aβ42_D7H_ aggregated into HMW assemblies in the no ion controls after 144 h. When Aβ42_wt_ or Aβ42_D7H_ was co-incubated with Zn^2+^_,_ we observed fewer HMW assemblies and more LMW assemblies than in the no ion control. However, when Aβ42_D7H_, but not Aβ42_wt_, was co-incubated with Cu^2+^_,_ we observed fewer HMW assemblies and more LMW assemblies than in the no ion control (Fig. 6J). The Western blot results are consistent with those of the ThT assay, indicating that the Aβ_D7H_ mutation shifts the sizes distribution of ion-induced Aβ oligomers into LMW assemblies.

For the TEM observations, Aβ was incubated with Zn^2+^ or Cu^2+^ in a 1∶1 ratio for 264–312 h. After incubation with Zn^2+^, we found that the Aβ_wt_ assemblies were predominantly annular protofibrils as we reported previously but the Aβ_D7H_ assemblies mostly had an amorphous morphology (Fig. 4). After incubation with Cu^2+^, the Aβ_wt_ assemblies were predominantly protofibrils and short fibrils. However, the Aβ_D7H_ assemblies were predominantly amorphous with occasional short fibrils (Fig. 4). The TEM results indicate that not only the size but also the morphology of ion-induced Aβ oligomers are altered by the D7H mutation.

### The D7H mutation promotes the interaction of Zn^2+^ and Cu^2+^ with Aβ

**Figure 7:**
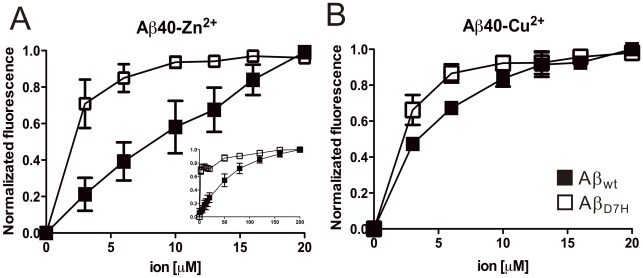
The D7H mutation promotes the binding of Zn^2+^ and Cu^2+^ to Aβ. The structural changes of 50 μM Aβ40_wt_ (▪) or Aβ40_D7H_ (□) during 0 to 20 μM Zn^2+^ (A) and Cu^2+^ (B) titration were monitored by 5 μM Bis-ANS. (A inlet) Aβ40_wt_ (▪) and Aβ40_D7H_ (□) were titrated by 0 to 200 μM Zn^2+^. The signals at 490 nm of Bis-ANS fluorescence were normalized and plotted to ion concentration. Data were presented as mean ± SEM from 3 independent experiments.

Our result suggests a higher susceptibility of Aβ_D7H_ to Zn^2+^/Cu^2+^ during aggregation process. To access a more direct evidence of Aβ-ion interaction, we used Bis-ANS [27], [28] to probe Aβ conformation at early aggregation stage in the presence or absence of ions to estimate the binding affinity of Aβ-ion complex. The 490 nm fluorescence signals of 50 μM Aβ in the presence of varying concentrations of Zn^2+^ or Cu^2+^ were collected. The final titration signal of each condition was used as unit for normalization (Fig. 7). Fluorescence signals without normalization are shown in figure S5. The Bis-ANS emission of Aβ40_wt_ and Aβ40_D7H_ had ∼6.5- and ∼11.5-fold increase in the presence of Zn^2+^ (Fig. S5A, C) but had ∼1.5- and ∼2.4-fold decrease in the presence of Cu^2+^ (Fig. S5B, D). Thus, at early aggregation stage, the D7H mutation exaggerated the ion-induced Aβ40 structural changes with Zn^2+^ increasing but Cu^2+^ decreasing exposure of hydrophobic clusters. For the Zn^2+^ titration, saturation of structural changes occurred at around 200 μM Zn^2+^ for Aβ40_wt_ (Zn^2+^: Aβ as 4∶1) and at around 5 μM Zn^2+^ for Aβ40_D7H_ (Zn^2+^: Aβ as 1∶10, Fig. 7A). For the Cu^2+^ titration, saturation of structural changes occurred at around 10 μM Cu^2+^ for Aβ40_wt_ (Cu^2+^: Aβ as 1∶5) and at around 5 μM Cu^2+^ for Aβ40_D7H_ (Cu^2+^: Aβ as 1∶10, Fig. 7B). Our result indicates that the D7H mutation promotes Aβ40 interaction with Zn^2+^ and Cu^2+^, where the Aβ interaction with Zn^2+^ is especially enhanced by the mutation.

### The D7H mutation has lower redox activity

**Figure 8:**
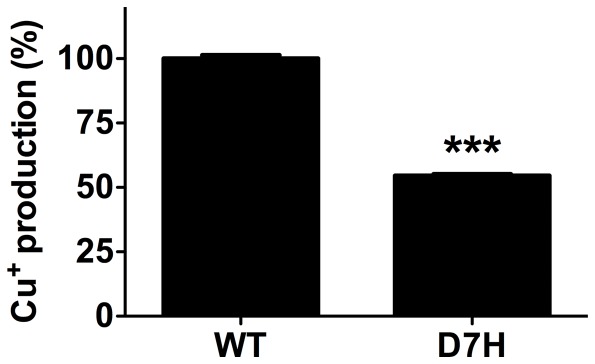
The D7H mutation decreases the redox activity of Aβ42 in metal reduction assay. Reduction of Cu^2+^ to Cu^+^ was performed by BCA assay. Freshly prepared 10 μM Aβ42_wt_ and Aβ42_D7H_ were mixed with BCA solution containing 4% CuSO_4_ to perform the redox activity assay. Data were presented as mean ± SEM (n = 3), ***P<0.0001.

The redox activity of Aβ has been suggested to play a role in neurotoxicity and oligomerization process. Altered redox activity may be one of the mechanisms underlying our findings. Thus, we examined the redox activity of Aβ42_wt_ and Aβ42_D7H_ by metal reduction assay with bicinchoninic acid (Fig. 8) [29]. The reaction provides a quantitative method for Cu^+^ production representing the capability of Aβ to reduce Cu^2+^ to Cu^+^. Our result demonstrated that Aβ42_D7H_ has ∼45% lower Cu^+^ production in comparison to that of Aβ42_wt_. The lower capability of Aβ42_D7H_ to reduce Cu^2+^ to Cu^+^ suggested a lower redox activity of Aβ42_D7H_ than Aβ42_wt_.

## Discussion

In this study, we report a novel intra-Aβ mutation, Aβ_D7H_, which has a “double punch” effect on the disease progress of AD by modulating both Aβproduction and oligomer formation.

APP overexpressing cell culture study indicated that the D7H mutation enhances the amyloidogenic cleavage pathway and raises Aβ production and the Aβ42/40 ratio. In vitro examination indicated that the D7H mutation shifts Aβ40 aggregation into the fibril-prone pathway and Aβ42 aggregation into the oligomer-prone pathway. According to the algorithm proposed by Guerreiro to classify AD mutations, we consider that this D7H mutation could be classified as “probably pathogenic” [26].

In addition, we characterized the biochemical features of Aβ_D7H_-ion complex, including the kinetic of fibril formation, size distribution, morphology and binding affinity. Our results of Aβ_wt_-ion are all compatible with others [27], [30], [31], [32]. Therefore, we provide an index of the biochemical features of Aβ-ion complex with a genetic hint, which might be more relevant to AD pathogenesis. Our study may contribute to the knowledge of designing Aβ-ion interrupting therapy in AD.

### The effect of intra-Aβ mutations on APP processing

Shifting APP processing into amyloidgenic pathway is one of the key factors in AD pathogenesis [24]. We speculated that the increase in Aβ levels and Aβ42/40 ratio of D7H mutant APP may accelerate Aβ accumulation in the brain. Usually, intra-Aβ mutations are less prone to interfere with APP processing. Only the A2V, E11K, and A21G mutations enhance amyloidogenic cleavage [8], [33], [34]. Interestingly, the D7N Tottori mutation does not affect Aβ levels or the Aβ42/40 ratio in the conditioned media of stably transfected N2a cells [14]. Besides the β-site cleavage to generate the C99 fragment, β-secretase could also cleave APP at the β′-site between Tyr10 and Glu11 to generate an 89 amino acid fragment (C89). The E11K mutation blocks the β′-site and shifts cleavage of APP to the β-site, causing increased Aβ production [8]. In this study, we did not detect significant differences in C89 level between wt APP and D7H mutant APP expressing cells, indicating that the D7H mutation does not interfere with β′ cleavage of APP.

Moreover, APP processing and trafficking could be regulated by imbalance of copper or zinc [35], [36], [37], [38]. Zn^2+^ and Cu^2+^ also bind to the E1 and E2 domain at N-terminal APP. Metal binding to E1 domain is related to the iron transport and APP ferroxidase-like activity [39]. Metal binding to E2 domain is suggested to relate with APP processing [40]. However, whether D7H changes metal binding to APP and alters APP structure, function and processing remains unclear. The mechanism by which D7H mutant APP favors the amyloidogenic cleavage pathway needs further investigation.

### The role of the Aβ N-terminal region in aggregation and toxicity

Similar to most of the intra-Aβ mutants, the Aβ42_D7H_ mutant induced more cell death than Aβ42_wt_, suggesting that the D7H mutation-induced aggregates are neurotoxic. The D7H mutation may increase toxicity through its effects on the duration of Aβ oligomer formation or on the structures of the aggregates thus formed. However, the SH-SY5Y cell might not be a good model of neurotoxic effect of Aβ as Aβ42_wt_ showing only a trend of toxicity (Fig. 5). For future studies, the pathological role of Aβ_D7H_ should be confirmed in primary culture, brain slices, or D7H mutant APP transgenic mice.

To our surprise, the D7H mutation had distinct effects on Aβ40 and Aβ42 fibrillization, which has not been reported for other intra-Aβ mutations. The fibrogenic properties of Aβ42 are signed by two additional residues, Ile41 and Ala42, altering its structure and hydrophobicity [41]. Nevertheless, an additional secondary structure between the Phe4-His14 region is found in Aβ40 but not Aβ42 fibrils [42]. The D7H mutation, which is located in this region, may have distinct effects on the aggregation properties of Aβ40 and Aβ42 by altering this N-terminal structure. This Aβ_D7H_ mutant provides an interesting tool for further biochemical study of the effect of the N-terminal region on the differential aggregation properties of Aβ40 and Aβ42.

### Effect of metal ions on Aβ aggregation

The high concentration of Zn^2+^ or Cu^2+^ in glutamatergic synapses has been proposed to promote Aβ aggregation and toxicity [21]. Interrupting Aβ-ion interaction with a metal-protein-attenuating compound, PBT2, has beneficial effects in the AD mouse model and in the phase II clinical trial [22]. We speculate that the pathogenicity of Aβ_D7H_ might be partially contributed by its higher affinity toward Zn^2+^/Cu^2+^ (Fig. 7). Consistent with this speculation, we show that the D7H mutation exaggerated the Zn^2+^/Cu^2+^-induced Aβ conformational changes (Fig. S5). The opposite effect of Zn^2+^ and Cu^2+^ on Aβ conformation at early aggregation stage has also been shown in our previous study [27].

Cu^2+^ has been shown to inhibit Aβ fibrillization and to induce assemblies with multiple morphologies [27], [43], [44]. The altered properties of Aβ_D7H_-Cu^2+^ complex might be the result of Cu^2+^ interaction with the additional histidines at Aβ position 7. Most of the free Cu^2+^ interacting with His6/His13 or His6/His14 promotes β-sheet-rich fibril formation, while a small proportion of Cu^2+^ interacting with the adjacent imidazole rings at His13/His14 inhibits fibril but promotes “amorphous” structure formation [44], [45]. Therefore, we speculate that the additional two adjacent imidazole rings at His6/His7 of Aβ_D7H_ promote the formation of “amorphous” non-β-sheet assemblies.

Zn^2+^ has been shown to inhibit fibril but to promote annular protofibril formation of Aβ_wt_ [27], [46]. In this study, Zn^2+^ promoted “amorphous” assemblies formation of Aβ_D7H_. Computational studies revealed that Asp7 is important for the stabilization of Zn^2+^-induced oligomers [47]. Therefore, we speculate that the loss of Asp7 in Aβ_D7H_ destabilize Zn^2+^-induced annular protofibril and thus promote “amorphous” aggregate formation. Together, our findings suggest that the “amorphous” aggregates induced by Zn^2+^/Cu^2+^ might be more relevant to AD pathology.

Mutations in 21^st^–23^rd^ residues of Aβ showed no differences in ion-induced aggregation while the ion-induced aggregation of Aβ N-terminus mutations has not been examined [48]. Our results provide the first genetic indication linking Zn^2+^ and Cu^2+^-induced Aβ aggregation to the pathogenesis of AD.

### Redox activity of Aβ

The histidine residues on Aβ are thought to play a role in controlling the redox activity of Cu^2+^ [49]. In our study, although Aβ_D7H_ had higher Cu^2+^ binding affinity (Fig. 7), Aβ_D7H_ had lower capability to reduce Cu^2+^ to Cu^+^ (Fig. 8). This indicates that the redox activity of Aβ-Cu^2+^ might be controlled by multiple factors rather than be simply controlled by the Cu^2+^ binding affinity. Redox activity has been suggested to involve in the Aβ-induced cytotoxicity and oligomerization [18], [50]. Lower redox activity of Aβ42_D7H_ suggested that redox activity is not the primary factor for Aβ42_D7H_-induced cytotoxicity. Furthermore, Aβ-Cu^+^ complex is suggested to promote cross-linking of peptides through dityrosine formation to stabilize oligomers [51], [52]. Nevertheless, Aβ42_D7H_ had lower Cu^+^ production (Fig. 8) but retained aggregates in LMW oligomers (Fig. 6), indicating that the Aβ42_D7H_ LMW oligomers might not be stabilized by dityrosine formation or redox activity. Together, the change in redox activity might not be the mechanism underlying our findings, but more details of redox activity other than copper reduction should be addressed.

## Methods

### Human subject and cell line information

This study was approved by Institutional Review Board at Taipei Veterans General Hospital. The written informed consent was obtained from the patient. The patient's guardian also consented on the behalf her because her capacity to consent was reduced. Human embryonic kidney (HEK293) cells were from Bioresource Collection and Research Center (60019, Hsinchu, Taiwan). SH-SY5Y human neuroblastoma cells were from Sigma-Aldrich (94030304, MO, USA).

### Materials

Metal ions were all prepared in double-distilled Mill-Q water. Purchasing information for all the materials used in this study is listed in supplementary materials (Method S1).

### Plasmid construction

cDNA encoding human wild-type hAPP770 was subcloned into a CMV promoter/enhancer-driven expression vector (pDEST26). A QuickChange II site-directed mutagenesis kit was used to introduce the D7H mutation into the wt APP construct. The correctness of the resulting constructs was confirmed by sequence analysis.

### Cell culture

Human embryonic kidney (HEK293) cells were transfected with the wt APP and the D7H mutant APP plasmids by Lipofectamin 2000 according to the manufacturer's protocol. 36 hours after transfection, cells were lysed with Trizol reagent to isolate total protein following the manufacturer's instruction.

### APP and Aβ measurement

To determine the levels of full length APP and the C-terminal fragments, 70 μg (Fig. 2A) and 30 μg (Fig. 2C) of cell lysates were separated by 15% Tris-Tricine SDS-PAGE and analyzed with a mouse anti-APP N-terminus antibody (22C11) or rabbit anti-APP C-terminus antibody (AB5352). To measure sAPPβ, conditioned media of APP expression cells were separated by 8% Tris-glycine SDS-PAGE and analyzed with a rabbit anti-sAPPβ antibody (9138-005). To monitor Aβ assemblies, the cross-linked samples were separated by 4%, 10%, and 15% stacking Tris-Tricine SDS-PAGE, and analyzed with an anti-Aβ 17–24 antibody (4G8). Human Aβ levels in APP transfected cells were quantitated by enzyme-linked immunosorbent assay (ELISA) using high sensitivity human β Amyloid 40 and 42 kits that use anti-Human Aβ 11–28 as the capture antibody. All antibodies used in this study do not recognize Aβ-Asp7 as an epitope.

### Aβ preparation

Aβ peptides were synthesized using Fmoc (N-(9-fluorenyl) methoxycarbonyl) chemistry and purified by reverse-phase high-performance liquid chromatography [53]. The molecular mass was identified by matrix-assisted laser desorption/ionization-time of flight (MALDI-TOF) mass spectrometry (UltraFlex II). For the Western blot, transmission electron microscopy (TEM) and 3-(4,5-Dimethylthiazol-2-yl)-2,5-diphenyltetrazolium bromide (MTT) assays, Aβ was prepared with hexafluoroisopropanol (HFIP) and dimethyl sulfoxide (DMSO) and incubated at room temperature for the indicated times [54]. For the thioflavin T (ThT) assay, Aβ were prepared with guanidine hydrochloride (GdnHCl) and incubated at 25°C for the indicated times [27], [54]. For the GdnHCl preps, lyophilized Aβ was dissolved in 8 M GdnHCl, incubated for 15–20 mins, and added to 10 mM Tris-HCl, pH 7.4 (GdnHCl: Tris buffer, 1∶9 v/v) for refolding. Impurities or aggregates were removed by centrifugation at 17,000×g for 20 min at 4°C. The supernatant was collected, and the Aβ concentration was determined by the absorbance at 280 nm (ε = 1,280 cm^−1^M^−1^) [55]. For the HFIP-DMSO preps, lyophilized Aβ was dissolved in HFIP and incubated for 1 h at room temperature. HFIP was removed by vacuum overnight. HFIP-treated Aβ films were dissolved in DMSO (Aβ: DMSO, 1∶100 w/v) and added to 10 mM Tris-HCl, pH 7.4 (DMSO: Tris, 1∶9 v/v). After centrifugation at 17,000×g at 4°C for 20 min, the supernatant was collected and quantified by absorbance at 280 nm.

### Photo-induced cross-linking of unmodified proteins (PICUP)

The experiment was performed as described previously [25], [27]. Briefly, 9 volumes of Aβ solution were mixed with 0.5 volume each of 1 mM Tris (2,2′-bipyridyl) dichlororuthenium(II) (RuBpy) and 20 mM ammonium persulfate. After mixing, the samples were exposed to a blue light LED in a closed chamber with a manual switch for 10 sec. The cross-linking reaction was stopped by adding SDS-PAGE sample buffer, and the samples were subjected to Tris-Tricine SDS-PAGE.

### ThT assay

25 μM of Aβ was incubated in 25 μM ThT at 25°C in an ELISA plate and monitored with a microplate reader. The ThT emission was measured at 485 nm, while excitation was at 442 nm. The signals were collected automatically every hour for 100 h.

### MTT assay

SH-SY5Y human neuroblastoma cells with ∼75% confluence were treated with Aβ_wt_ or Aβ_D7H_ for 48 hours at 37°C. After 48 hours of incubation, the MTT was added, and the cultures were incubated for an additional 3 h. Cells were lysed overnight using a lysis buffer containing 10% SDS and 20 mM HCl. The absorbance was measured at a wavelength of 570 nm by an ELISA reader.

### Transmission electron microscopy (TEM)

10 μl of 25 μM Aβ samples was placed on glow-discharged, 400-mesh formvar carbon-coated copper grids, negatively stained with 2% uranyl acetate, and examined with a TEM with an accelerating voltage of 75 kV.

### Ion titration and Bis-ANS fluorescence

Fluorescence emission spectra of 4,4-Bis (1-anilinonaphthalene 8-sulfonate) (Bis-ANS) were collected at wavelengths ranging from 450 to 550 nm with an excitation wavelength of 400 nm. 50 μM Aβ in 5 μM Bis-ANS was titrated with 520 μM ZnCl_2_ or CuCl_2_ to final ion concentrations in the range of 0–20 μM and with 6640 μM ZnCl_2_ to final ion concentrations in the range of 20–200 μM at 25°C in a circulating water bath. The total volume was increased by less than 10% after titration. The signals at 490 nm were used for normalization. The changes of each titration signal to the initial titration signal were normalized to the change of the final titration signal to the initial titration signal. The normalized data were plotted against metal ion concentration.

### Metal reduction assay

Aβ42_wt_ and Aβ42_D7H_ were prepared in the HFIP-DMSO preps. The metal reduction assay was performed as described previously [29] by using bicinchoninic acid (BCA) assay kit. The working solutions were freshly prepared following the manufacturer's manual. Briefly, the working solution, 200 μl, was added to polystyrene 96-well plate with transparent bottom and 25 μl of Aβ42_wt_ and Aβ42_D7H_ with final concentrations at 10 μM were added into the wells. The plate was then incubated at 37°C and read continuously at absorbance of 562 nm by SpectraMax M5 Multi-Mode microplate reader to monitor Cu^+^ production. The absorption was generated from the BCA-Cu^+^ complex. The saturated absorption at 120 min were obtained, subtracted by the buffer control, averaged (n = 3), and normalize to the intensity obtained from Aβ42_wt_.

## Supporting Information

Figure S1 Pedigree and laboratory data. (A) The pedigree of a Taiwanese family with early onset of AD. The index patient is indicated by an *. The family members with AD are labeled in black. (B) The early-onset AD patient showed normal laboratory data in complete blood count, liver function, thyroid function, renal function and syphilis. This excluded other possibilities from neurodegenerative diseases.
(TIF)

Figure S2 Transfect efficiency and APP maturity of wt APP and D7H mutant APP. HEK293 cells were transfected with 0.8 μg pDEST26 plasmid encoding either wt APP or D7H mutant APP. (A) After 24 h, number of cells transfected with APP was analyzed by a mouse anti-APP N-terminus antibody (22C11, red) and number of cell were estimated by DAPI staining (blue). Transfection efficiencies for both plasmids were ∼20%. Scale bar: 50 μm. (B) After 36 h, 30 μg of cell lysates were separated by 8% SDS-PAGEs. APP was analyzed with a mouse anti-APP N-terminus antibody (22C11) and actin was served as loading control. APP expression level was similar in wt APP and D7H mutant APP expressing cells. (C) APPs were separated by 8% SDS-PAGEs and analyzed by anti-APP N-terminus antibody (22C11). Graph showing the fold change of the ratio of mature/immature APP indicates that the ratio of APP maturity of wt APP and D7H mutant APP is similar. Data from wt APP expressing cells were set as 1 in 3 independent experiments and presented as mean ± SEM.
(TIF)

Figure S3 The D7H mutation did not alter intracellular Aβ level. ELISA showed no significant increase in ratios of Aβ40/APP, Aβ42/APP and Aβ42/40 in the cell lysate of wt APP and D7H mutant APP transfected cells. Data from wt APP expressing cells were set as 1 in 3 independent experiments and presented as mean ± SEM.
(TIF)

Figure S4 Different Aβ preparations also confirmed that the D7H mutation promotes Aβ40 HMW assemblies but promotes Aβ42 LMW assemblies formation. (A, B) Lyophilized Aβ40 (A) and Aβ42 (B) were prepared in HFIP-DMSO for the ThT assay. Data were averaged from 3–4 independent experiments. (C, D) Lyophilized Aβ40 (C) and Aβ42 (D) were prepared in HFIP-DMSO for Western blotting without PICUP treatment.
(TIF)

Figure S5 The representative emission spectra of Aβ40_wt_ (A, B) or Aβ40_D7H_ (C, D) in the presence of 0, 10, 20, and 50 μM Zn^2+^ (A, C) or Cu^2+^ (B, D) are shown.
(TIF)

Method S1 Human embryonic kidney (HEK293) cells were from Bioresource Collection and Research Center (60019, Hsinchu, Taiwan). SH-SY5Y human neuroblastoma cells were from Sigma-Aldrich (94030304, MO, USA). MALDI-TOF mass spectrometry was produced by Bruker BioSciences (Bruker Daltonics Ultraflex, MA, USA). The microplate reader for the ThT assay and the BCA assay was produced by Molecule Devices (SpectraMax M5, CA, USA). TEM was produced by Hitachi (H-7000, Tokyo, Japan). Fluorescence microscope was produced by ZEISS (Axio Observer A1, Ireland). Spectrofluorometer for binding affinity was produced by Horiba Jobin Yvon (FluoroMax-3, USA). Copper grids for TEM were purchased from EMS Inc. (18086, PA, USA). Lipofectamin 2000 and pDEST26 were from Invitrogen (11809-019 and 11668-500, USA). The site-directed mutagenesis kit was from Stratagene (200521, CA, USA). Antibody 22C11, AB5352 and 4G8 were from Millipore (MAB348, AB5352 and MAB1561, MA, USA). Antibody for sAPPβ was from Convance (9138-005). Antibody for β-actin was from GeneTex (GTX110564, CA, USA). Mounting medium with DAPI was from by Vector Laboratoies (H-1200, CA, USA). ELISA kits for human Aβ40 and Aβ42 were purchased from Wako (294-62501 and 290-62601, Japan). GdnHCl was from Merck (1.04220.1000, Darmstadt, Germany). ThT, Trizol, 1,1,1,3,3,3-Hexafluoro-2-propanol (HFIP), γ-secretase inhibitor (L-685,458), Tris (2,2′-bipyridyl) dichlororuthenium (II) (Ru(Bpy)), CuCl_2_ and ZnCl_2_ were purchased from Sigma-Aldrich (T3516, T9424, 105228, SI-L1790, 224758, 12317 and 31650, MO, USA). Tris and ammonium persulfate (APS) were from Amresco (0826 and 0486, OH, USA). 3-(4,5-dimethylthiazol-2-yl)-2,5-diphenyltetrazolium bromide (MTT) was from Bio Basic Inc. (298-93-1, Taipei, Taiwan). ELISA reader was produced by SUNRISE, TECAN (Switzerland). The bicinchoninic acid (BCA) assay kit was from Thermo Scientific (Waltham, MA, United States). The polystyrene 96-well plate used for BCA assay was from UltraViolet (Taipei, Taiwan).
(DOC)
