# Supplementary figures and images for: Amyloid-Beta (Aβ) D7H Mutation Increases Oligomeric Aβ42 and Alters Properties of Aβ-Zinc/Copper Assemblies
Source: PLoS One. 2012 Apr 30;7(4):e35807. doi: 10.1371/journal.pone.0035807 (PMC3340413; doi:10.1371/journal.pone.0035807)

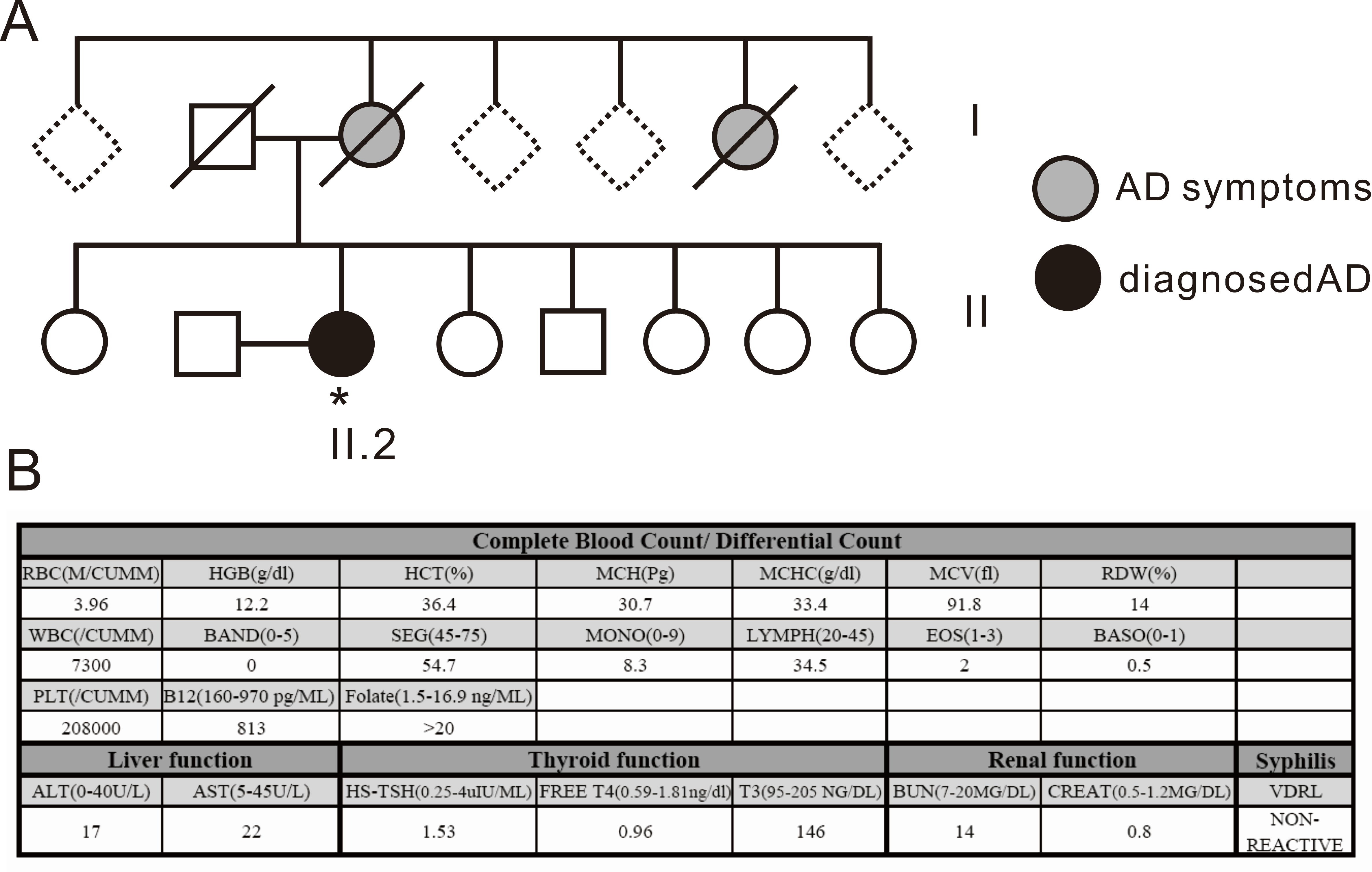

Supplement: Figure S1 — Pedigree and laboratory data. (A) The pedigree of a Taiwanese family with early onset of AD. The index patient is indicated by an *. The family members with AD are labeled in black. (B) The early-onset AD patient showed normal laboratory data in complete blood count, liver function, thyroid function, renal function and syphilis. This excluded other possibilities from neurodegenerative diseases. (TIF) [file pone.0035807.s001.tif]

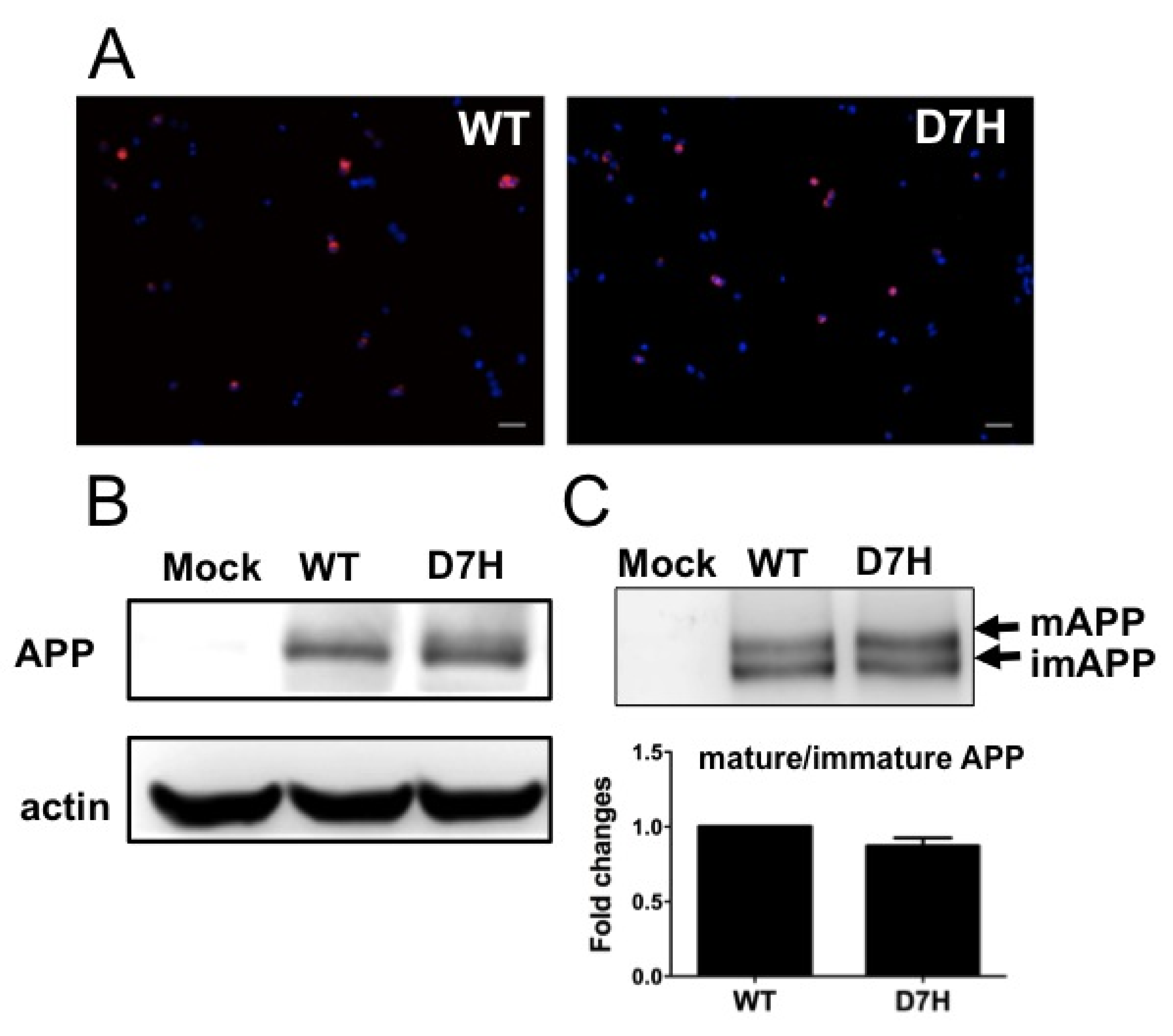

Supplement: Figure S2 — Transfect efficiency and APP maturity of wt APP and D7H mutant APP. HEK293 cells were transfected with 0.8 μg pDEST26 plasmid encoding either wt APP or D7H mutant APP. (A) After 24 h, number of cells transfected with APP was analyzed by a mouse anti-APP N-terminus antibody (22C11, red) and number of cell were estimated by DAPI staining (blue). Transfection efficiencies for both plasmids were ∼20%. Scale bar: 50 μm. (B) After 36 h, 30 μg of cell lysates were separated by 8% SDS-PAGEs. APP was analyzed with a mouse anti-APP N-terminus antibody (22C11) and actin was served as loading control. APP expression level was similar in wt APP and D7H mutant APP expressing cells. (C) APPs were separated by 8% SDS-PAGEs and analyzed by anti-APP N-terminus antibody (22C11). Graph showing the fold change of the ratio of mature/immature APP indicates that the ratio of APP maturity of wt APP and D7H mutant APP is similar. Data from wt APP expressing cells were set as 1 in 3 independent experiments and presented as mean ± SEM. (TIF) [file pone.0035807.s002.tif]

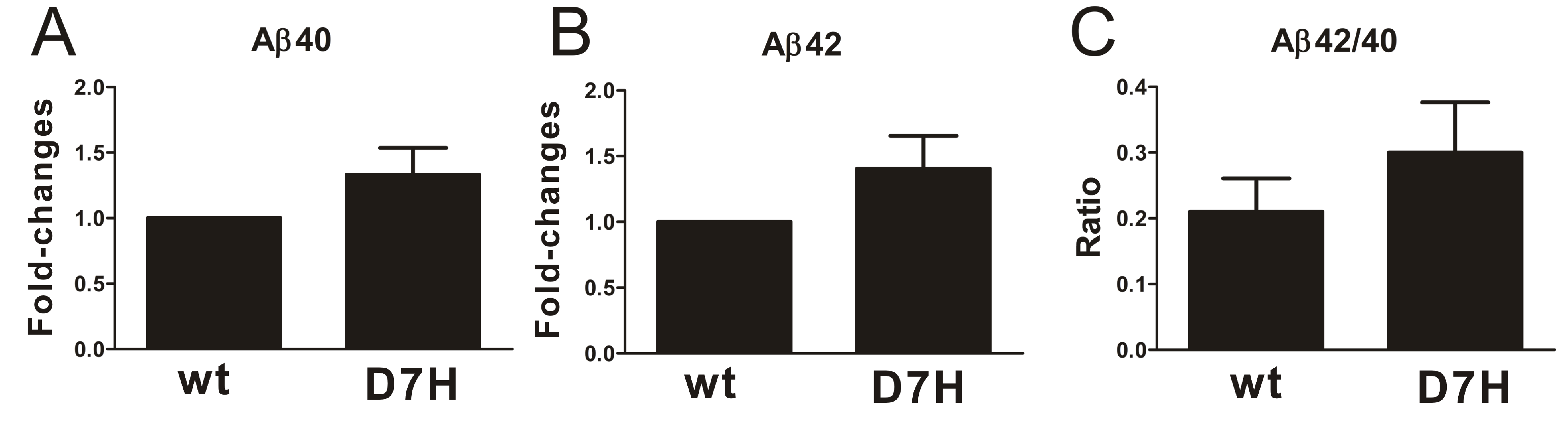

Supplement: Figure S3 — The D7H mutation did not alter intracellular Aβ level. ELISA showed no significant increase in ratios of Aβ40/APP, Aβ42/APP and Aβ42/40 in the cell lysate of wt APP and D7H mutant APP transfected cells. Data from wt APP expressing cells were set as 1 in 3 independent experiments and presented as mean ± SEM. (TIF) [file pone.0035807.s003.tif]

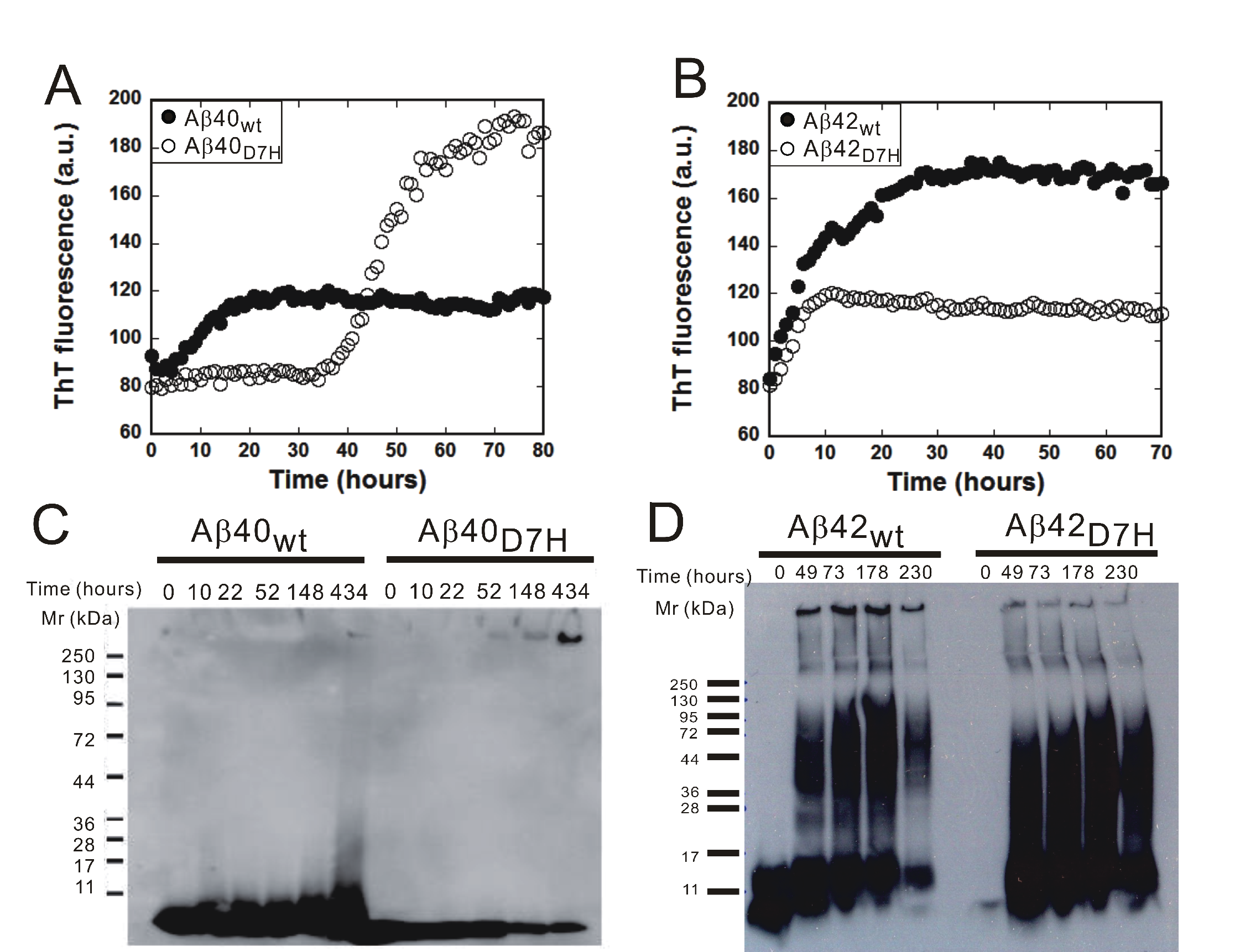

Supplement: Figure S4 — Different Aβ preparations also confirmed that the D7H mutation promotes Aβ40 HMW assemblies but promotes Aβ42 LMW assemblies formation. (A, B) Lyophilized Aβ40 (A) and Aβ42 (B) were prepared in HFIP-DMSO for the ThT assay. Data were averaged from 3–4 independent experiments. (C, D) Lyophilized Aβ40 (C) and Aβ42 (D) were prepared in HFIP-DMSO for Western blotting without PICUP treatment. (TIF) [file pone.0035807.s004.tif]

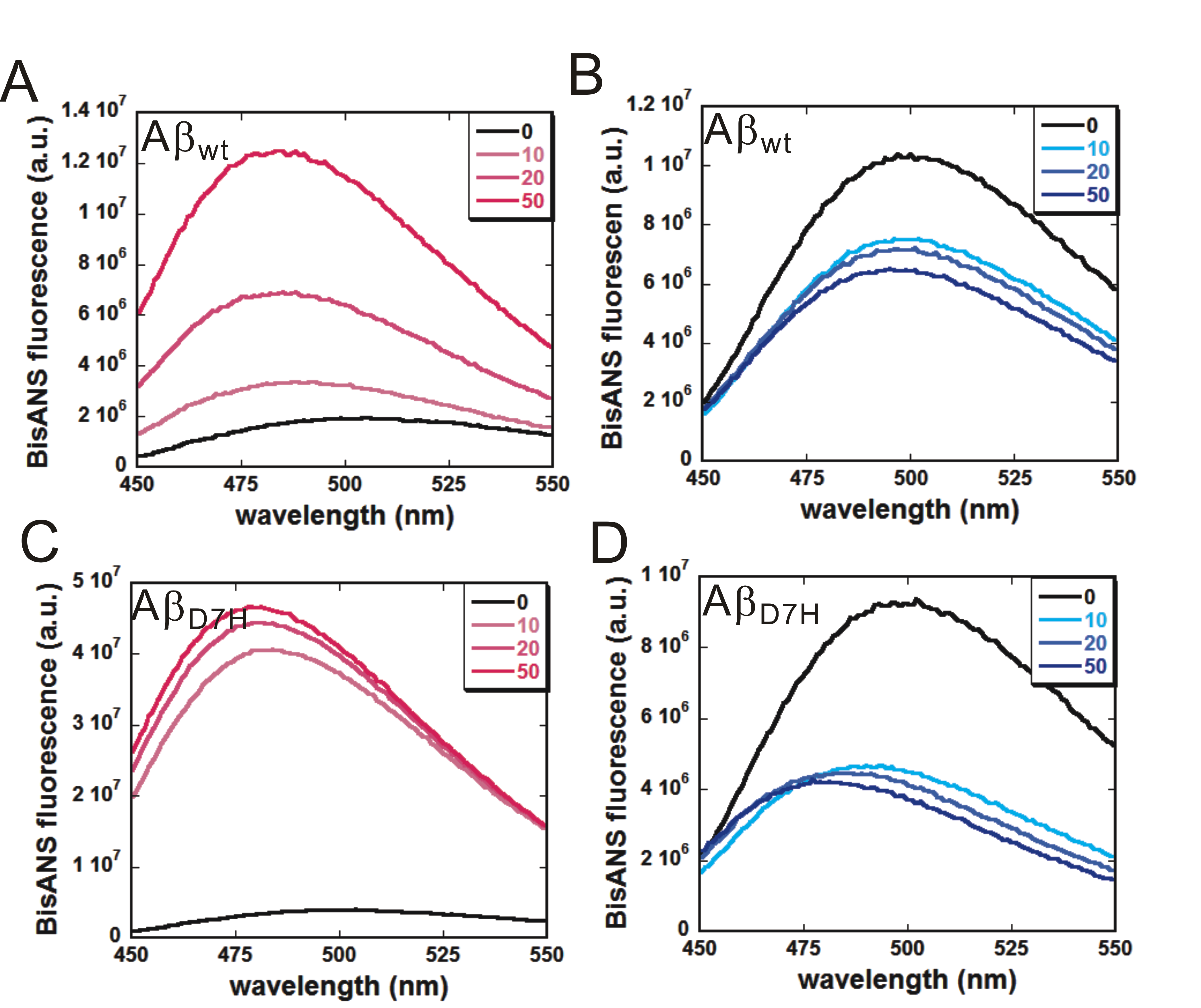

Supplement: Figure S5 — The representative emission spectra of Aβ40wt (A, B) or Aβ40D7H (C, D) in the presence of 0, 10, 20, and 50 μM Zn2+ (A, C) or Cu2+ (B, D) are shown. (TIF) [file pone.0035807.s005.tif]
