# Supplementary material for: Amyloid-Beta (Aβ) D7H Mutation Increases Oligomeric Aβ42 and Alters Properties of Aβ-Zinc/Copper Assemblies
Source: PLoS One. 2012 Apr 30;7(4):e35807. doi: 10.1371/journal.pone.0035807 (PMC3340413; doi:10.1371/journal.pone.0035807)
Supplement: Method S1 — Human embryonic kidney (HEK293) cells were from Bioresource Collection and Research Center (60019, Hsinchu, Taiwan). SH-SY5Y human neuroblastoma cells were from Sigma-Aldrich (94030304, MO, USA). MALDI-TOF mass spectrometry was produced by Bruker BioSciences (Bruker Daltonics Ultraflex, MA, USA). The microplate reader for the ThT assay and the BCA assay was produced by Molecule Devices (SpectraMax M5, CA, USA). TEM was produced by Hitachi (H-7000, Tokyo, Japan). Fluorescence microscope was produced by ZEISS (Axio Observer A1, Ireland). Spectrofluorometer for binding affinity was produced by Horiba Jobin Yvon (FluoroMax-3, USA). Copper grids for TEM were purchased from EMS Inc. (18086, PA, USA). Lipofectamin 2000 and pDEST26 were from Invitrogen (11809-019 and 11668-500, USA). The site-directed mutagenesis kit was from Stratagene (200521, CA, USA). Antibody 22C11, AB5352 and 4G8 were from Millipore (MAB348, AB5352 and MAB1561, MA, USA). Antibody for sAPPβ was from Convance (9138-005). Antibody for β-actin was from GeneTex (GTX110564, CA, USA). Mounting medium with DAPI was from by Vector Laboratoies (H-1200, CA, USA). ELISA kits for human Aβ40 and Aβ42 were purchased from Wako (294-62501 and 290-62601, Japan). GdnHCl was from Merck (1.04220.1000, Darmstadt, Germany). ThT, Trizol, 1,1,1,3,3,3-Hexafluoro-2-propanol (HFIP), γ-secretase inhibitor (L-685,458), Tris (2,2′-bipyridyl) dichlororuthenium (II) (Ru(Bpy)), CuCl2 and ZnCl2 were purchased from Sigma-Aldrich (T3516, T9424, 105228, SI-L1790, 224758, 12317 and 31650, MO, USA). Tris and ammonium persulfate (APS) were from Amresco (0826 and 0486, OH, USA). 3-(4,5-dimethylthiazol-2-yl)-2,5-diphenyltetrazolium bromide (MTT) was from Bio Basic Inc. (298-93-1, Taipei, Taiwan). ELISA reader was produced by SUNRISE, TECAN (Switzerland). The bicinchoninic acid (BCA) assay kit was from Thermo Scientific (Waltham, MA, United States). The polystyrene 96-well plate used for BCA assay was from UltraViolet (Taipei, Taiwan). (DOC) [file pone.0035807.s006.doc]

**Supplemental Material and Method**

**Materials** Human embryonic kidney (HEK293) cells were from Bioresource Collection and Research Center (60019, Hsinchu, Taiwan). SH-SY5Y human neuroblastoma cells were from Sigma-Aldrich (94030304, MO, USA). MALDI-TOF mass spectrometry was produced by Bruker BioSciences (Bruker Daltonics Ultraflex, MA, USA). The microplate reader for the ThT assay and the BCA assay was produced by Molecule Devices (SpectraMax M5, CA, USA). TEM was produced by Hitachi (H-7000, Tokyo, Japan). Fluorescence microscope was produced by ZEISS (Axio Observer A1, Ireland). Spectrofluorometer for binding affinity was produced by Horiba Jobin Yvon (FluoroMax-3, USA). Copper grids for TEM were purchased from EMS Inc. (18086, PA, USA). Lipofectamin 2000 and pDEST26 were from Invitrogen (11809-019 and 11668-500, USA). The site-directed mutagenesis kit was from Stratagene (200521, CA, USA). Antibody 22C11, AB5352 and 4G8 were from Millipore (MAB348, AB5352 and MAB1561, MA, USA). Antibody for sAPPβ was from Convance (9138-005). Antibody for β-actin was from GeneTex (GTX110564, CA, USA). Mounting medium with DAPI was from by Vector Laboratoies (H-1200, CA, USA). ELISA kits for human Aβ40 and Aβ42 were purchased from Wako (294-62501 and 290-62601, Japan). GdnHCl was from Merck (1.04220.1000, Darmstadt, Germany). ThT, Trizol, 1,1,1,3,3,3-Hexafluoro-2-propanol (HFIP), γ-secretase inhibitor (L-685,458), Tris (2,2’-bipyridyl) dichlororuthenium (II) (Ru(Bpy)), CuCl_2_ and ZnCl_2_ were purchased from Sigma-Aldrich (T3516, T9424, 105228, SI-L1790, 224758, 12317 and 31650, MO, USA). Tris and ammonium persulfate (APS) were from Amresco (0826 and 0486, OH, USA). 3-(4,5-dimethylthiazol-2-yl)-2,5-diphenyltetrazolium bromide (MTT) was from Bio Basic Inc. (298-93-1, Taipei, Taiwan). ELISA reader was produced by SUNRISE, TECAN (Switzerland). The bicinchoninic acid (BCA) assay kit was from Thermo Scientific (Waltham, MA, United States). The polystyrene 96-well plate used for BCA assay was from UltraViolet (Taipei, Taiwan).
